# Classifying epilepsy pragmatically: Past, present, and future

**DOI:** 10.1016/j.jns.2021.117515

**Published:** 2021-05-29

**Authors:** Nathan A. Shlobin, Gagandeep Singh, Charles R. Newton, Josemir W. Sander

**Affiliations:** a Department of Neurological Surgery, Feinberg School of Medicine, Northwestern University, Chicago, IL, USA; b Department of Neurology, Dayanand Medical College, Ludhiana, India; c NIHR University College London Hospitals Biomedical Research Centre, UCL Queen Square Institute of Neurology, Queen Square, London WC1N 3BG, UK; d Department of Psychiatry, University of Oxford, Oxford, UK; e Kenya Medical Research Institute, Kilifi, Kenya; f Stichting Epilepsie InstelClingen Nederland (SEIN), Heemstede, the Netherlands; 7 Chalfont Centre for Epilepsy, Chalfont St Peter SL9 0RJ, UK

**Keywords:** Integrated epilepsy classification, 2017 ILAE classification, Four-dimensional epilepsy classification, Epilepsy

## Abstract

The classification of epilepsy is essential for people with epilepsy and their families, healthcare providers, physicians and researchers. The International League Against Epilepsy proposed updated seizure and epilepsy classifications in 2017, while another four-dimensional epilepsy classification was updated in 2019. An Integrated Epilepsy Classification system was proposed in 2020. Existing classifications, however, lack consideration of important pragmatic factors relevant to the day-to-day life of people with epilepsy and stakeholders. Despite promising developments, consideration of comorbidities in brain development, genetic causes, and environmental triggers of epilepsy remains largely user-dependent in existing classifications. Demographics of epilepsy have changed over time, while existing classification schemes exhibit caveats. A pragmatic classification scheme should incorporate these factors to provide a nuanced classification. Validation across disparate contexts will ensure widespread applicability and ease of use. A team-based approach may simplify communication between healthcare personnel, while an individual-centred perspective may empower people with epilepsy. Together, incorporating these elements into a modern but pragmatic classification scheme may ensure optimal care for people with epilepsy by emphasising cohesiveness among its myriad users. Technological advancements such as 7T MRI, next-generation sequencing, and artificial intelligence may affect future classification efforts.

## 1. Introduction

Epilepsy classification is essential for people with epilepsy, caregivers, healthcare personnel, researchers, policymakers, and insurers [1,2]. Classification allows people with epilepsy to identify with a well-defined condition, empower them, and provide a direction to engage with others. Classification schemes also clarify communication, enhancing care and augmenting education and training [1–3].

The first classification of seizures and epilepsies was conceived in 1964 and popularised in the 1970’s [2,4,5]. Classifications have been published under the aegis of the International league Against Epilepsy (ILAE) with the most recent updates in 2017 [2,6,7]. In 2001, a multidimensional classification scheme with five axes [8] and in 2012, a four-dimensional classification scheme was proposed [9,10]. An Integrated Epilepsy Classification (IEC) scheme based on commonalities between the ILAE and four-dimensional classification schemes was proposed [11]. Classification continues to evolve while enduring lively debates and having been criticised for its focus on detail and at the same time, lack of inclusiveness and pragmatism [12].

Given the importance of classification, it is crucial to consider the need for further refinement and adaptability. We revisit past and present milestones in epilepsy classification, review past and existing classifications, propose future considerations, and examine rising technologies’ influence. This review provides consideration of previously undervalued factors influencing the debate on the ever-evolving epilepsy classification.

## 2. Past and current classifications

ILAE published in 1981 its first seizure classification after the development of video-EEG monitoring (Table 1) [13,14]. Seizures were divided into partial and generalised according to onset. The 1985 epilepsies classification scheme was based on semiology with age dependency and etiology in addition to EEG features [14,15]. ILAE revised its proposal in 1989 with categories including localisation-related epilepsies and syndromes, generalised epilepsies and syndromes, undetermined syndromes and special syndromes [14,16]. Contemporaneously, an ILAE expert group also proposed an epidemiologic classification of epilepsies [17]. A diagnostic scheme proposed in 2001 involved five axes mirroring those of the DSM IV: ictal phenomenology, seizure type, syndrome, etiology, and impairment [8,14]. The 2006 update of this scheme further delineated self-limited epilepsy syndromes [1,14,18]. Another proposal (2010) incorporated the concept of brain networks between subcortical and cortical structures and between cortical areas [14,19]. Non-mutually exclusive etiological classifications such as genetic, structural, metabolic, and unknown were created [14,19]. ILAE Commissions generated new classifications for seizures and epilepsies in 2017 (ILAE-EC) involving seizure type, epilepsy types, and etiologies [6,7,14,20].

**Fig. 1:**
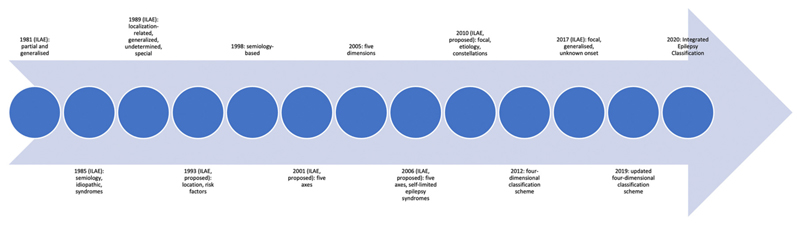
Timeline of past and current classifications.

A group of experts proposed a classification entirely based on seizure semiology in 1998 [21–23]. They offered a five-dimensional individual-oriented classification scheme consisting epileptogenic zone location, seizure semiology, etiology, seizure frequency, and related conditions in 2005 [24]. A four-dimensional classification (4D-EC) system consisting of semiology of the seizures, epileptogenic zone location, etiology, and associated comorbidities was proposed in 2012 [9,10]. The updated four-dimensional classification scheme (4D-CS) comprises a sequential approach to categorising non-specific paroxysmal events [10]. The current ILAE classification and 4D-CS were merged into the IEC after considering similarities between the two [11]. The IEC contains five subcategories: header, seizure type, epilepsy type, etiology, and comorbidities and relevant individual preferences [11]. Fig. 1 demonstrates a timeline of past and current classifications.

## 3. The past and present classifications

The intense debate on epilepsy classification is reassuring. Numerous proposals and refutations notwithstanding, the discussions bear out experts’ engagement in the evolution of classification. Limitations, however, have arisen at each step. Some stem from the semantics, syntax and semiotics of seizures. For instance, the term dialeptic seizures has been used in the past to emphasise the phenomenon of behavioural arrest observed in absence seizures and mesial temporal seizures [25]. The term dyscognitive seizures has also been used with diverse connotations by experts, only to be removed in a later version of the classification [6,7,14,20]. Descriptive terminology for mesial temporal seizures has evolved from complex partial seizures to dialeptic seizures, dyscognitive seizures, and focal seizures with or without impaired awareness [6]. The variety of expressions can confuse beginners and prove challenging for the experienced to relearn [25,26]. To the credit of the creators, it is now accepted that any classification should be flexible to meet the needs of different users. There is also a perceived need to explore beyond the boundaries of current classifications to encompass horizons yet not covered.

### 3.1. Caveats in classifications

Mutually exclusive etiological categories lead to confusion. For example, GLUT1 transporter deficiency may be denoted as genetic or metabolic [11], while neurocysticercosis may be classified as infective or structural. These ambiguities could be addressed by specifying etiological conditions precisely [11]. The classification of a specific condition may, however, have implications for the estimation of the burden of disease. Additionally, classifications have removed anatomical origins due to the imperfect relationship between location and semiology and the electro-clinical similarity between seizures arising from different lobes [1]. The character of seizures, however, bear a relationship to a lobe [27,28]. The ILAE, 4D-EC, and IEC classifications also include syndromes with differing importance [6,7,10,11]. Several population and facility-based studies have examined the yield of the syndromic classification. In these studies (Table 2), syndromes were unambiguously assigned in 4–97% of cases [29–35]. The variation represents the differences between samples assessed, methodologies used and experts’ experience [36–38].

### 3.2. Comorbidities

People with epilepsy are up to eight times more likely to have conditions such as depression, anxiety, migraine, heart disease, peptic ulcers, and arthritis relative to the general population [39], and more likely to have other neuropsychiatric disorders, pain disorders, autoimmune diseases and asthma [40,41]. The presence of comorbidities, however, must be assessed across different populations to gain acceptance [41,42]. There seems to be a biological basis for the association of epilepsy with psychosomatic comorbidities [43–47]. Similarly, existing methods of quantifying comorbidities such as the Charlson Comorbidity Index and Elixhauser Comorbidity Index are generally unsuitable for epilepsy [42]. An epilepsy-specific risk adjustment index may be necessary to account for comorbidities properly [42].

Inclusion of comorbidities into classification should be expanded. The 2017 ILAE classification includes comorbidities for the first time [48]. The 4D-CS and IEC included comorbidities, and the IEC asks users to list comorbidities relevant to the individual [9–11]. The incorporation of comorbidities merits further consideration given the occurrence and treatment of comorbidities influences the expression, treatment, and outcome, and vice-versa. Systematic classification of comorbidities should involve creating a master list of known comorbidities while suiting the differing needs of the treatment providers of epilepsy and the comorbidities. Uniform documentation of associated comorbidities diagnoses will permit the identification of appropriate treatments, increase consideration of the impact of comorbidities in epilepsy presentation and its treatment, and improve communication between different specialists.

### 3.3. Changes in the demography of epilepsy

While current classifications presume a static nature to populations, epilepsy demography has changed. An appropriate representation of these trends may provide a clearer categorisation of individuals with epilepsy.

First, temporal trends are important to examine. Epilepsy incidence is high in the first year of life, perhaps due to to the high proportion of symptomatic cases presenting early in life [49]. Many of these children have epileptic encephalopathies, often with psychomotor arrest that predisposes developmental slowing and seizures later in life [49]. Other children have generalised or focal seizures of the neonatal or infantile period [49]. Medical technology has also contributed to the high first-year incidence as premature children and children with congenital anomalies or severe early life insults survive longer with a higher likelihood of developing epilepsy. Incidence declines by the end of the first decade [50,51]. Decreased exposure to teratogens including some antiseizure medications (ASMs) and environmental risk factors may have enhanced the decrease [52,53]. The incidence increases in the elderly due to cerebrovascular diseases, neurodegenerative disorders, intracerebral tumors, and traumatic brain injury [54–56], although standardisation account for this [55–57]. Second, consideration of changes in the etiology of epilepsy is required. The concept of etiology of epilepsy has changed over time as conceptual models of causality in epilepsy continue to be developed and risk factors continue to be elucidated [58]. Third, the state of epilepsy is relevant, Whether a patient has active epilepsy or epilepsy in remission, controlled or uncontrolled epilepsy, and drug-resistant epilepsy influences the prognosis [59–61]. An adequate epilepsy classification must capture these distinctions to add relevance to clinical practice.

Incorporating the changing demographics of epilepsy will improve communication between specialists, allowing for the greater utility of classification schemes by emphasising the most affected groups.

### 3.4. Brain age

Brain development stages denoted as “brain age”, measured often as brain-predicted age difference (PAD) [62,63], is probably more relevant than chronological age to epilepsy diagnosis and classification, influencing susceptibility to, presentation, and consequences of seizures. The onset or offset of seizures marks a turning point in brain development, demonstrated by regression of speech with seizure onset in Kleffner-Landau syndrome or resumption of brain maturation with successful seizure control in West syndrome [64,65]. In epileptic encephalopathies, brain maturation arrests at a given time point and might also regress. Therefore, consideration of how brain age or development is reflected in classification is important.

Developing, aging, or degenerating brains are highly susceptible to seizures [66,67]. Brain maturation likely influences seizure semiology. Seizures are featureless, denoted as “hypomotor”, during infancy, plausibly reflecting limited neuronal connectivity, while automatisms and hypermotor seizures occur in older children with presumably mature brains [68]. Lateralizing signs increase with age in people with temporal epilepsy [69]. Epileptic spasms typically occur during infancy, while absence seizures, myoclonic-astatic, and generalised tonic-clonic seizures occur in later childhood [70,71]. Lastly, there is evidence for an interaction between age and pharmacoresistance to ASMs, adversely impacting cognitive development and function in children with epilepsy. Cognitive impairments associated with uncontrolled seizures are particularly severe during infancy and decrease thereafter [72,73]. Individuals with onset of temporal epilepsy in childhood exhibit greater reduction of brain tissue volumes, namely white matter in extratemporal regions, and more marked memory deficits [74].

While the quest for robust markers for brain age continues, it is conceivable that applications of artificial intelligence and machine learning will yield important insights to admit brain age to epilepsy classifications in the future.

### 3.5. Genetic etiologies

**Fig. 2:**
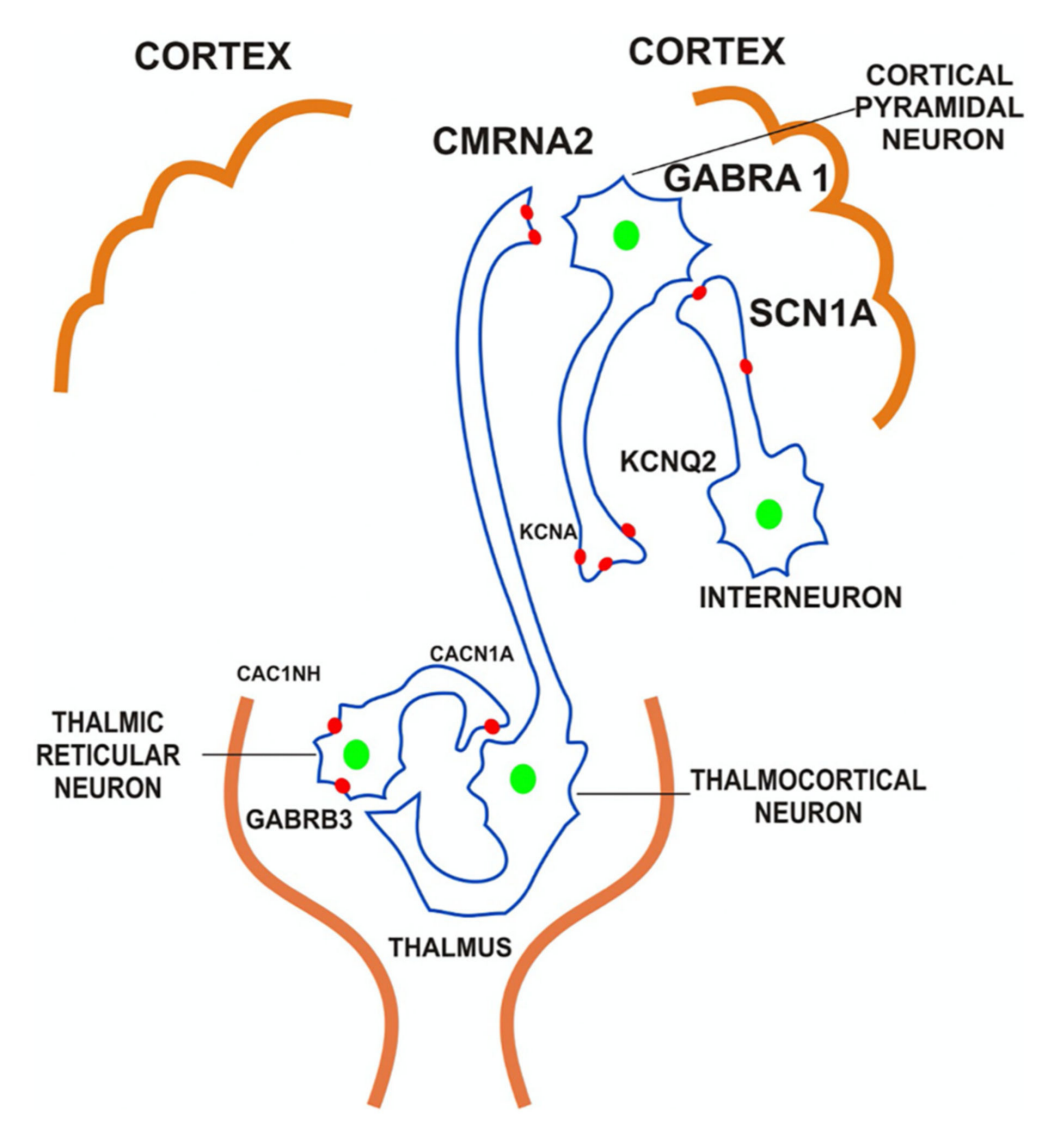
Channels commonly affected in epilepsy.

Current classification paradigms incorporate genetic etiologies, but there exists little description of the specific genetic characteristics associated with the diagnosis. Polygenic theory suggests that an accumulation of single nucleotide polymorphisms associated with epilepsy may explain the propensity of certain individuals to develop epilepsy [75]. Conversely, pathogenic variants lead to epilepsy development through several mechanisms. Discovery of genes contributing to epilepsy is rapidly growing. Myriad single gene disorders have been implicated in epilepsy (Table 3) [47,76,77]. Common mechanisms include voltage-gated channelopathies [78–81], ligand-gated channe-lopathies [79,80,82–86], neurotransmitter release machinery [79,87,88], and structural alterations (Table 4) [79,89]. Fig. 2 demonstrates commonly affected channels. Delineation of genetic attributes promotes research to elucidate the natural history of the condition, leads to the development of precision medications, guides treatment paradigms, facilitates preventative measures, and helps individuals with a genetic disorder connect with each other.

As genetic links for epilepsy are increasingly uncovered, conceptualising classification in terms of genetic causation becomes indispensable. The 4D-EC and IEC permit the use of genetic information, given their emphasis on providing as much detail as available [9–11], but existing classification schemes do not intentionally incorporate genetic etiologies. Investigations such as chromosomal microarrays, whole genome and whole exome sequencing, and gene panels are now increasingly available. These investigations secure a genetic diagnosis and aid in the syndromic and etiological classification despite the cost and access issues. Specific syndromes benefit from contemporary genetic testing. These include epilepsies developing before the age of 2, especially epileptic encephalopathies, suspected and imaging-confirmed brain malformations, and certain inborn errors of metabolism and selected syndromes such as West Syndrome and Dravet Syndrome [90–93]. Undoubtedly, the elucidation of a genetic diagnosis is likely to influence classification elements and systems in the future.

### 3.6. Environmental triggers

Environmental factors might contribute to susceptibility to and development of epilepsy. Febrile infections herald fever-related syndromes [94,95]. Malnutrition lowers seizure threshold perhaps through hyponatremia, or hypocalcemia [96,97]. Traumatic brain injury may trigger seizures through GABA signaling disinhibition [98,99]. Photosensitivity and altered circadian rhythms may lead to seizures through altered sensory integration [100,101]. Other environmental triggers include various prenatal and postnatal factors [102], though these must be elucidated in further human studies.

Environmental and genetic factors possibly interact to trigger epilepsy [102]. Environmental stimuli may be required to express genes involved in epilepsy or enhance the effect of the susceptibility genotype [102]. This effect appears to differ between acquired and the so-called “genetic epilepsies”. Genetic events influence acquired epilepsies, and the genetic epilepsies are modified by acquired factors [103]. Given their epilepsies role, ion channels may be a mechanism involved in the gene-environment interaction [103]. Environmental and genetic factors may synergistically alter the density, stoichiometry, and post-translational modification of the same ion channels [103]. Acknowledging ecological factors and gene-environment interactions in future classification schemes will allow for greater representation of the etiology and targeted management of people with epilepsy.

## 4. Future epilepsy classifications

Current classifications fulfill clinical needs through their applicability and adaptability in allowing certain epilepsies to be labelled as unknown. Dimensions that may improve the precision of discussions between and address various users of classification are lacking. Cross-contextual validation of the classification and emphasis on a teambased and individual-centred care are required to develop a comprehensive conceptualisation of care.

### 4.1. Validation of the classification cross-contextually

Any classification scheme must be applicable to contexts differing in socioeconomic factors, cultures, and practice settings. This is the case in epilepsy as approximately 80% of people with epilepsy live in low-and-middle-income countries (LMICs) [104]. Three-fourths of them do not receive appropriate treatment [105,106]. There is a dearth of epilepsy specialists in LMICs [105–107], so people receive care mainly through primary care, if any. They experience markedly higher premature mortality [108]. Hierarchies of importance placing epilepsy below other chronic conditions also contribute to a greater burden of epilepsy in LMICs.

Epilepsy classification is central in tackling the treatment gap and mortality burden in LMICs. Infectious diseases such as neurocysticercosis, malaria, and encephalitis are common in LMICs, and hence, valid case definitions linking these to epilepsies must be applied [109,110]. Local variations in culturally-specific conceptualisations, manifestations, and epilepsy effects must be incorporated into classification schemes [111]. A study in rural China determined a substantial portion of generalised epilepsy previously characterised were labelled unknown upon the release of the 2017 ILAE-EC [35]. Forms of epilepsy common in LMICs differ from those in high income countries due to unique but often multiple risk factors [112]. Classification systems must be adaptable to different settings by all care providers to communicate effectively [105–107]. Clinicians must prioritise the needs of their population in classifying epilepsy to guide resource allocation [110]. ILAE has provided basic and advanced versions of classifications, but a singular classification scheme with flexibility to address local needs will enable public health efforts and policy [110]. It is also essential to allow classification with minimal or no use of technology [110]. Characteristics such as age of onset, semiology, family history, risk factors, treatment response, and relevant comorbidities can be assessed in clinics [110]. Even in LMICs, classifications should enable flexibility to identify locally relevant factors and resource constraints. These efforts can be coupled with capacity-building with field workers, increased availability of low-cost technology such as telemedicine, and public education campaigns to promote and provide appropriate treatment [113–116]. Ensuring broad applicability of the classification will include more people with epilepsy globally, thereby providing them with proper treatment and help close the treatment gap [105,106].

### 4.2. Comprehensive team-based approach to epilepsy

A classification system must be coupled with a comprehensive team-based approach to care, research, and policymaking. Specialists create current classifications. Involving primary health care workers such as physicians, nurses, and ancillary staff, as well as researchers, policymakers, and other parties, into discussions will prove productive [117–119]. This will allow for appropriate refinement of specific terms used to describe seizures, create a glossary of key terms with associated definitions, and resolve discrepancies and ambiguities [11]. The classification system must then be clarified to all personnel to increase their understanding [120,121].

Given the need for team-based approach, an elucidation of users’ needs of classification is warranted. Table 5 shows a list of potential users of an epilepsy classification and different levels of informational needs to accommodate all parties involved. Incorporating levels of descriptiveness into the classification will ensure that the communication-related needs of all individuals involved are met. The headline portion should be emphasised as the “lingua franca” among health-care personnel with varying experience, care centers, and across socio-cultural contexts [11]. A linkage to the previous classification systems to preserve continuity and allow for monitoring of trends is also necessary [120]. Professional organisations and educational bodies within existing health-care structures can have primary responsibility in encouraging adoption of the classification [122,123].

### 4.3. Person-centered care

It is important to recognise the centrality of people with epilepsy in efforts to refine classifications. Any classification system must be explained to people and family members or caregivers and incorporate mechanisms to obtain feedback during development. This will increase understanding of the condition, connect with the care team through greater trust and self-involvement in care through acquiring additional information and engaging in self-advocacy [117–119,121]. A classification system must involve consideration of person-centred outcomes and the needs of individuals in addition to traditional measures of disease status [124,125]. Measures of health-related quality of life and personal impact should be acquired to assess how epilepsy or ASMs are impacting individuals [125]. This will guide clinicians regarding possible changes to the frequency of clinic visits, event monitoring or medication regimen or alert them of the need for referral to other physicians. Lastly, the classification scheme should emphasise a holistic approach to care [126]. Epilepsy poses a large logistical and psychological burden to individuals [126–128]. Younger people may experience feelings of apprehension regarding revealing their diagnosis, while age-related metabolic changes often burden adults, cognitive decline, increased risk for seizure-related injuries, extensive comorbidities, and polypharmacy [126,129]. Depression, anxiety, and a lack of social connection with other individuals are common [126,129]. Provision of care appropriate to the specific concerns of people with epilepsy, psychosocial interventions to increase self-efficacy and locus of control, and measures to enhance social support may empower people with epilepsy [127,128].

## 5. Technologies likely to impact future classifications

Consideration of the effect of seven Tesla (7 T) magnetic resonance (MRI), next-generation sequencing (NGS), and artificial intelligence and machine learning on classification is required to reduce the fraction of unknown epilepsies and enhance the versatility of classification as future technological developments change clinical practice.

### 5.1. 7T MRI

The 2017 ILAE classification scheme reclassified 27% of generalised and 7% of focal cryptogenic epilepsies into epilepsies of unknown type in one study [130]. There is a need to reduce the proportion of unknown epilepsies further. Seven Tesla MRI with increased spatial resolution allowing visualisation of internal structures and differentiation of pathological tissue from normal tissue might conceivably help in reducing this proportion [131–134]. 7 T provides for detection of lesions previously undiscovered [135–137]. Unaided review of 7 T images reveals previously unseen lesions in 22% of cases, while utilising a morphometric analysis program raises this proportion to 43% [135]. 7 T-morphometric analysis uncovers a quarter more lesions than 3 T morphometrics [135].

Additionally, 7 T allows characterisation of focal cortical dysplasia and hippocampal sclerosis and volumetric analysis of epilepsy-related brain regions [131,132,138–141]. The efficacy of 7T MRI in epilepsy classification relative to 3T MRI is yet to be fully assessed. The 4D-EC and IEC allow for incorporating imaging findings [9–11], but there is no concerted effort. However, the increased utilisation of 7 T may enable precise classification by distinguishing epilepsy types and etiologies, thereby reducing the proportion of unknown epilepsies.

### 5.2. Genome sequencing

NGS has markedly increased the speed of genome sequencing [142–144]. The ability of NGS to find causal mutations, including de novo mutations, associated with epilepsy syndromes enhances molecular diagnosis [145]. NGS is beneficial to identify genetic causes in people with earlier seizure onset, and a family history [146,147]. Currently, available gene panels exhibit substantial variability, ascertaining up to 265 genes with reported diagnostic yields up to 48.5% [148]. NGS may be unable to determine the precise genetic etiology for epilepsies with polygenic inheritance [149] but its utility extends beyond genetic factors. NGS uncovers inherited metabolic disorders in 13% of people with normal metabolic investigations [150]. NGS has the potential to refine metabolic, infective, and autoimmune causes by identifying genetic alterations associated with these etiological categories. Similarly, NGS enhances understanding of pathogenesis through genotype-phenotype correlations, allowing for refined diagnosis [151]. Through the inclusion of genes associated with epilepsy and the possibility of discovering novel mutations, greater adoption of NGS may improve classification by comprehensively characterising genetic factors, catalysing reclassification of unknown epilepsies into well-delineated categories.

### 5.3. Artificial intelligence and machine learning

The use of artificial intelligence and machine learning in epilepsy has grown substantially [152,153]. Artificial neural networks have been utilised in tandem with multiwavelet transform techniques to diagnose epilepsy with high accuracy, sensitivity, and specificity based on EEG data [154–159]. Artificial intelligence and machine learning have also been used to localize seizure onset zones from EEG data [160–164]. For example, an unsupervised algorithm can collate the localisation of epileptiform discharges over a day into a single map [164]. Recording periods of less than two hours may enable clinically meaningful characterisation of seizure onset zone [162]. Recently, artificial intelligence and machine learning approaches have examined seizure classification [165–169]. A text mining approach based on ICD-9 yielded good performance in detecting complex focal seizure, simple focal seizure, and convulsive epilepsy based on data from the electronic medical record [166]. It is possible to distinguish temporal from extratemporal seizure by extracting spatiotemporal features from facial and pose semiology from EEG-records [167]. Studies analysing EEG data with multiple extraction methods have found high accuracy, sensitivity, and specificity [168,169]. Development of capabilities to differentiate a more significant number of seizure types, identify associated pathology and probable etiology, and characterise epilepsies based on multimodal inputs may enable delineation of previously unrecognised factors to clarify ambiguities in classification, create additional classes, and reduce the proportion of unknowns.

## 6. Conclusions

Epilepsy classification is evolving with promising recent developments. Incorporating stages of brain development, genetic and environmental triggers, and changes in the demography into a modernised classification is necessary. Validation of this classification in different socioeconomic status contexts and coupling with a team-based approach and person-centred perspective is also required. These factors may ensure optimal care by addressing increasing the ease and precision of communication between the myriad of individuals who utilise the epilepsy classification. Technological advances, including 7T MRI, genome sequencing, and artificial intelligence, may prove helpful in improving future epilepsy classification.

## Figures and Tables

**Table 1:** Existing epilepsy classification schemes.

Organization	Name	Year	Salient features
ILAE	ILAE	1981	Partial: simple, complex, secondary Generalised: absence, myoclonic, clonic, tonic-clonic, tonic
		1985	Semiology: focal vs. generalised Idiopathic: idiopathic vs. symptomatic Epilepsies are syndromes
		1989	Localization-related epilepsies and syndromes, generalised epilepsies and syndromes, epilepsies and syndromes undetermined to be generalised or focal, and special syndromes
		1993 (proposed)	Location: Generalised, partial, multiple seizure types, and unclassified seizures Risk factors: provoked, unprovoked seizure of unknown etiology, cryptogenic
		2001 (proposed)	Five axes: ictal phenomenology, seizure type, syndrome, etiology, impairment
		2006 (proposed)	Five axes: ictal phenomenology, seizure type, syndrome, etiology, impairment Delineation of self-limited epilepsy syndromes
		2010 (proposed)	Focal replaced partial Etiology; genetic, structural, metabolic, unknown Constellations: electroclinical syndromes with specific combinations of semiological, radiological, or pathological findings
		2017	Focal: networks limited to one hemisphere Generalised: engage both hemispheres but begin anywhere within generalised networks Seizures of unknown onset: if more information required Epilepsies sequentially classified by seizure type, epilepsy type, and epileptic syndromes
Other	Semiology	1998	Semiology-based: auras, autonomic seizures, dialeptic seizures, motor seizures, special seizures
	Five dimensional	2005	Five dimensions: location of epileptogenic zone, seizure semiology, etiology, seizure frequency, related medical conditions
	4D-CS	2012	Four dimensions: semiology, location of epileptogenic zone, etiology, associated comorbidities
		2019	Epileptic paroxysmal events: semiology, location of epileptogenic zone, etiology of epilepsy, associated comorbidities Non-epileptic paroxysmal events: organic or psychogenic
	IEC	2020	Headline, seizure type, epilepsy type, etiology, comorbidities / relevant individual preferences

Integrated Epilepsy Classification (IEC), International League Against Epilepsy (ILAE), four-dimensional classification scheme (4D-CS).

**Table 2:** Studies describing the yield of ILAE syndromic classifications.

Type of Study	Study	Country	Classifier(s)	Findings
Population-based	Oka et al., 2006	Japan	Neurologist	• 15.2% of people with epilepsy were classified into syndromic categories
	Olafsson et al., 2005	Iceland	Neurologist	• 58% of cases fell into non-informative categories
	Wang et al., 2019	China	Neurologist	• Unknown epilepsy increased from 1.2% with 1985 ILAE classification to 2.8% with 2017 ILAE classification
Primary care-based	Murthy et al., 1998	India	Neurologists	• 48% of people with epilepsy fell into ILAE categories
Tertiary care centrebased	Manford et al., 1992	United Kingdom	Epileptologist	• 33.6% of people with epilepsy were in diagnostic ILAE categories
	Kellinghaus et al., 2004	United States	Epileptologist	• 4% of adults and 21% of children were diagnosed with specific epilepsy syndrome
	Gao et al., 2018	China	Neurologist	• 44.5% of cases were not classified with 1981 ILAE classification, while 7% of cases were not classified with the 2017 ILAE classification

**Table 3:** Single gene disorders implicated in epilepsy.

Condition	Genes
Angelman syndrome	*UBE3A*
Aristaless-relaxed homeobox gene (ARX) disorders	*ARX*
Autosomal dominant epilepsy with auditory features	*LG11*
Autosomal dominant juvenile myoclonic epilepsy	*GABRA1, CACNB4, CLCN2*
Autosomal dominant nocturnal frontal lobe epilepsy	*CHNRA4, CHNRNB2*
Benign familial neonatal convulsion	*KCNQ2, KCNQ3*
Benign familial neonatal-infantile seizures	*SCN2A*
Dravet syndrome	*SCN1A*
Early onset absence epilepsy	*SLC2A1*
Generalised epilepsy with febrile seizures plus	*SCN1A, SCN2A, SCN2B, GABRG2*
Hot water reflex epilepsy	*SLC1A1*
Juvenile myoclonic epilepsy type 1	*EFHC1*
Lafora body disease	*EMP2A, NHLRC1*
Myoclonic epilepsy with ragged-red fibers (MERRF)	*TK, TL1, TH, TS1*
Neurofibromatosis	*NF1, NF2*
Neuronal ceroid-lipofuscinoses / Batten disease	*CLN3, CLN5, TPP1*
Protocadherin-19 (PCDH 19) related epilepsy	*PCDH-19*
Rett syndrome	*MECP2*
Severe myoclonic epilepsy of infancy	*SCN1A*
Sialidosis	*NEU1, PCDH19*
Tuberous sclerosis	*TSC1, TSC2*
Unverricht-Lundborg myoclonus epilepsy	*CTSB*

**Table 4:** Common genetic mechanisms for the development of epilepsy.

Category	Component	Mechanism
Voltage-gated channelopathies	Na^+^ channel	Inappropriate activation of current Prolonging activation Incomplete activation of channels Acceleration of recovery from inactivation
	K^+^ channel	Prolong neuronal depolarization through slow deactivation, loss of high-frequency bursting, or prolongation of membrane repolarization
	Ca^2+^ channel	Promote neuron synchrony by lowering thresholds for electrogenesis
Ligand-gated channelopathies	GABA channel	Reduction of GABA-activated Cl-current Increase in rate of desensitization
	Nicotinic ACh receptor	Slowed desensitization
	NMDA glutamate receptor	Increased duration of excitation
	AMPA glutamate receptor	Initiating excitation
	Metabotrobic glutamate receptor	Blockade of accommodation to a steady current Potentiation of effects of NMDA, AMPA, and depolarization
	Serotonin receptor	Loss of inhibitory current
Neurotransmitter release machinery	Synapsins 1 and 2	Decreased size of presynaptic vesicle pool particularly in inhibitory synapses
	Sv2A	Sustained release of neurotransmitters
	Vesicular zinc sequestration	Neuron hypersynchrony
	Reduced recycling	Prolonging activation
Structural	Cortical dysplasias	Inhibited postnatal granule cell proliferation in dentate gyrus Hypertrophy of neocortex Cell migration, segmentation, and patterning reduced Inhibitory neurons reduced or inhibited

**Table 5:** Potential Users of Epilepsy Classification.

Informational Need	User	Role
Sufficient knowledge conveyed in a comprehensible manner	People with epilepsy	Understand condition, treatments, and prognosis; care for oneself; connect with other people with a similar condition
	Family members / caregivers	Understand condition, treatments, and prognosis; care for their family member; join support groups for family members / caregivers of people with epilepsy
Technical information to provide monitoring and basic care	Electroneurodiagnostic technicians Nurses	Acquiring context for EEG outputs Acquire relevant information and convey accurate information to physicians
Specialized descriptions to determine management and provide referrals when appropriate	Primary care physician	Manage the everyday care of people with epilepsy and know when to refer to an epilepsy specialist
Further technical information for complex epilepsy care	Neurologist	Diagnose and manage the epilepsy-specific care of people with epilepsy; know when to refer to epilepsy specialist
Precise classification language to localize and manage epilepsy	Epilepsy specialist	Diagnose and manage the epilepsy-specific care of people with epilepsy
medically or surgically	Neurosurgeon	Decide whether surgical management is warranted, select the surgical technique, and perform surgery
Sufficient knowledge to conduct studies	Genetics researcher	Understand the genetics, phenotypic expressions, and variations in both with regard to epilepsy
Clear descriptions to guide research	Public health researcher	Understanding the epidemiology and outcomes of types of epilepsy
	Pharmaceutical manufacturer	Understanding which types of epilepsy require development or refinement of antiseizure medications
Precise delineation of conditions to guide financing and policy	Insurer	Understand how to determine reimbursement for epilepsy care
	Funding authority	Determine funding priorities for epilepsy research
	Policymaker	Understanding the burden and economic consequences of epilepsy
